# Transverse colon varices 

**Published:** 2022

**Authors:** Behzad Hatami, Naghmeh Salarieh, Pardis Ketabi Moghadam, Mehran Mahdavi, Azam Farahani

**Affiliations:** 1 *Gastroenterology and Liver Diseases Research Center, Research Institute for Gastroenterology and Liver Diseases, Shahid Beheshti University of Medical Sciences, Tehran, Iran*; 2 *Taleghani Hospital, Shahid Beheshti University of Medical Sciences, Tehran, Iran*

**Keywords:** Colonic varices, Band ligation, BRTO

## Abstract

Portal hypertension associated with liver cirrhosis usually leads to gastroesophageal varices; however, ectopic varices secondary to liver cirrhosis are not common, especially colonic varices which occur with a low frequency.

We are going to discuss the case of a 75-year-old man with liver (HBV) cirrhosis who was admitted to the hospital with rectorrhagia. Colonoscopy revealed evidence of acute bleeding in tortuous colonic varices. The band ligation performed during the colonoscopy had failed to control the bleeding.

The patient was referred to Taleghani Hospital in Tehran, and rectorrhagia was subsequently successfully controlled by BRTO technique (balloon-occluded retrograded transvenous obliteration).

## Introduction

 Colonic varices are collaterals between the portal and systemic circulation that extend above the dentate line in the rectum. In colonoscopy, they are considered as dilated tortuous submucosal veins dark blue in color (1). Almost all patients with colonic varices present with lower gastrointestinal hemorrhage which is often massive (2). The incidence of colonic varices is not known, but it is estimated to be very rare. Only two cases were reported among 2912 adult autopsies (3). No specific treatment or guidelines for colonic varicose veins have been determined (1) (4). 

## Case report

A 72-year-old man with a history of hepatitis B cirrhosis presented with rectorrhagia at the emergency room of Qazvin Hospital. EGD was performed. Two columns of grade 1-2 esophageal varices with no red signs along with portal hypertensive gastropathy were noticed on upper endoscopy. On colonoscopy, tortuous varices were noted in the transverse colon with fresh blood in the lumen. Band ligation was performed on transverse colon varices during the colonoscopy.

Upon admission to the emergency department of Taleghani Hospital, the patient was alert and oriented. On physical examination, his blood pressure was 100/70, pulse rate was 100 bpm, saturation without supplemental oxygen was 98%, and temperature was 36.5 ^º^C.

The patient had jaundice and pale conjunctiva. Abdominal examination revealed soft abdomen with mild distension. Rectal assessment showed fresh blood.

Laboratory tests disclosed HB: 6.4 g/dl, plt: 80000×10mm^3^, albumin: 2.8g/dL, bilirubin total: 4, bilirubin direct: 2, AST: 32, ALT: 26, international normalized ratio (INR): 1.4, and prothrombin time: 34.

**Figure 1:**
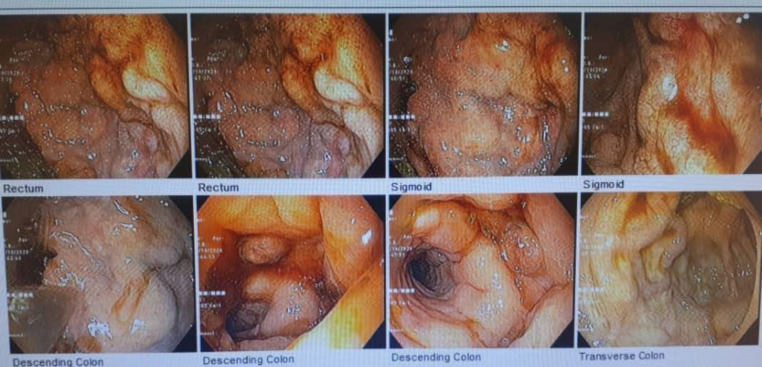
Colonic varices

**Figure 2:**
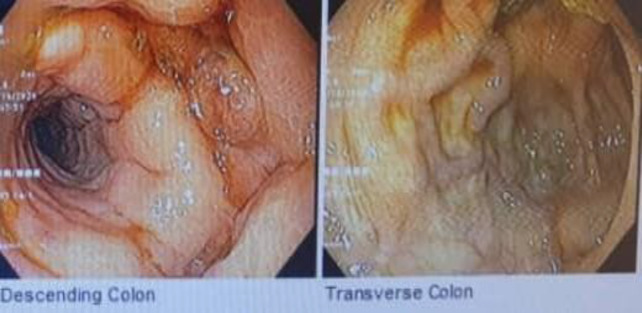
*T* *ransverse* *colon*

A colonoscopy was performed, and multiple tortuous varices were seen from the anal verge up to the transverse colon associated with clots without active bleeding (Figures 1, 2).

Intravenous octreotide (50 mcg/h) and multiple units of packed red blood cells and fresh frozen plasma were transfused.

The patient’s hemoglobin increased to 8.5 gr/dl, and he remained hemodynamically stable for the next few days.

An abdominal CT scan with IV contrast was done and revealed partial thrombosis in the extrahepatic portal vein expanding to the superior mesenteric vein.

Eventually, the patient underwent balloon occluded retrograde transvenous obliteration (BRTO). He remained hemodynamically stable after the procedure.

## Discussion

The main method for diagnosing rectal varices is endoscopy. Blue tinted elevations located near the anus that are related to portal hypertension are the most familiar feature of rectal varices. One study estimated that regardless of etiology, colonic varices have a prevalence rate of only 0.07% (5); in patients with cirrhosis it increases to 1% to 8% (6, 7). The most common sites of colonic varices are the rectum and the cecum (8). 

Although colonoscopy is the principal method for diagnosing colon varices, with massive bleeding, its diagnostic rate is 69%, because the varices may be obscured by blood (9). Thus, in cases of massive hematochezia, MRI, abdominal CT scan, and mesenteric angiography are alternative diagnostic tools.

Similar to esophageal varices, colonic varices are classified into four groups: F0: no varices; F1: small and straight varices; F2: enlarged and tortuous varices; and F3: large and coil-shaped varices. The colors are known as white (Cw) or blue (Cb). Red color signs like small vessels and telangiectasias can be seen as well (1) (10). In the current case, colonic varices were F3Cb and extended from the rectum to the transverse colon, which is very rare.

Management of bleeding in colonic varices, like other types of GI bleeding, is achieved through hemodynamic resuscitation and coagulopathy correction.

Resuscitation means repletion of intravascular volume and maintaining hemoglobin levels at approximately 8 gr/dl. The first step was done appropriately in our patient, and prophylactic antibiotic was commenced as well. Different methods have been proposed to control bleeding from colon varicose veins, but there is no specific treatment. Guideline suggestions include:

- Endoscopy methods: sclerotherapy, cyanoacrylate injection, band ligation.
- Surgical management: portocaval shunt surgery, simple suture ligation.
- Radiology intervention: TIPS, BRTO, embolization (11).

Endoscopic management methods, such as sclerotherapy and endoscopic band ligation, have been useful procedures in these patients; however, they were not successful in the current case.

An endovascular technique which causes occlusion of the outflow portosystemic shunt using an occlusion balloon (BRTO) is another available procedure for these patients (1). It is less invasive than transjugular intrahepatic portosystemic shunt (TIPS) and can be performed in patients with low residual liver (12). BRTO showed some advantages over TIPS in managing the acute phase. 

Finally, BRTO was performed on the current case, and fortunately it appears to have been successful. The first successful BRTO was done in 2006 on descending colon varices. Anan et al. reported a case of colonic varice in a cirrhotic patient with encephalopathy that was successfully treated by BRTO (13).

Chantal et al. reported the case of a 55-year-old man with alcoholic liver cirrhosis and prerectal bleeding due to cecal varices that were treated by BRTO (14).

A limited number of studies have been conducted on colon varicose veins.

## Conflict of interests

The authors declare that they have no conflict of interest.

## References

[1] Al Khalloufi K, Laiyemo AO (2015). Management of rectal varices in portal hypertension. World J Hepatol.

[2] Gudjonsson H, Zeiler D, Gamelli RL, Kaye MD (1986). Colonic varices Report of an unusual case diagnosed by radionuclide scanning, with review of the literature. Gastroenterology.

[3] FeldmaN M Sr, Smith VM, Warner CG (1962). Varices of the colon Report of three cases. JAMA.

[4] Kitagawa S, Sato T, Hirayama A (2015). Colonic Varices Due to Chronic Pancreatitis: A Rare Cause of Lower Gastrointestinal Bleeding. ACG Case Rep J.

[5] McCormack TT, Bailey HR, Simms JM, Johnson AG (1984). Rectal varices are not piles. Br J Surg.

[6] Ganguly S, Sarin SK, Bhatia V, Lahoti D (1995). The prevalence and spectrum of colonic lesions in patients with cirrhotic and noncirrhotic portal hypertension. Hepatology.

[7] Hosking SW, Smart HL, Johnson AG, Triger DR (1989). Anorectal varices, haemorrhoids, and portal hypertension. Lancet.

[8] Sato T, Akaike J, Toyota J, Karino Y, Ohmura T (2011). Clinicopathological features and treatment of ectopic varices with portal hypertension. Int J Hepatol.

[9] Abraham-Igwe C, Patel R (2002). Idiopathic colonic varices: a case report. Endoscopy.

[10] Idezuki Y (1995). General rules for recording endoscopic findings of esophagogastric varices (1991). Japanese Society for Portal Hypertension. World J Surg.

[11] Kanagawa H, Mima S, Kouyama H, Gotoh K, Uchida T, Okuda K (1996). Treatment of gastric fundal varices by balloon-occluded retrograde transvenous obliteration. J Gastroenterol Hepatol.

[12] Saad WE (2012). Balloon-occluded retrograde transvenous obliteration of gastric varices: concept, basic techniques, and outcomes. Semin Intervent Radiol,.

[13] Anan A, Irie M, Watanabe H, Sohda T, Iwata K, Suzuki N (2006). Colonic varices treated by balloon-occluded retrograde transvenous obliteration in a cirrhotic patient with encephalopathy: a case report. Gastrointest Endosc.

[14] Liu C, Srinivasan S, Babu SB, Chung R (2020). Balloon-occluded retrograde transvenous obliteration of colonic varices: a case report. CVIR Endovasc.

